# Outcomes of robotically assisted laparoscopic lateral suspension (RALLS) with mesh for anterior and apical prolapse

**DOI:** 10.1007/s11701-021-01234-3

**Published:** 2021-04-05

**Authors:** Patrick Dällenbach, Milena Alec, Michel Boulvain, Stas Shabanov

**Affiliations:** Urogynecology Unit, Division of Gynecology, Department of Pediatrics, Gynecology and Obstetrics, Geneva University Hospitals, 30 boulevard de la Cluse, 1211 Geneva 14, Switzerland

**Keywords:** Pelvic organ prolapse, Sacrocolpopexy, Hysteropexy, Apical prolapse, Robotic surgery, Lateral suspension with mesh

## Abstract

Abdominal sacral colpopexy/hysteropexy is the gold standard for the treatment of vaginal apex support. However, dissection of the promontory may expose to potentially life-threatening complications. To avoid this risk, laparoscopic lateral suspension with mesh is an alternative. Robotic assistance may be helpful in both techniques. The objective of our study was to evaluate outcomes of robotically assisted laparoscopic lateral suspension (RALLS) with mesh for anterior and apical pelvic organ prolapse (POP). From March 2012 to January 2018, 59 consecutive patients underwent RALLS using titanized polypropylene mesh. Between August 2017 and September 2019, all patients were contacted to assess outcome. We performed a clinical exam and asked them to complete the patient global impression of improvement (PGI-I) questionnaire. Fifty-four patients (91.5%) were available for follow-up. Mean age was 58.5 years (28.8–79.8). There were no perioperative complications. The mean follow-up was 33.6 months (11.2–74.1).The objective cure rate (no prolapse beyond hymen) and the subjective cure rate (PGI-I ≤ 2) were 83.3% and 77.2%, respectively. Five women (9.3%) were reoperated for POP recurrence*.* There was no erosion. Of the 20 women complaining of stress urinary incontinence (SUI) preoperatively, 12 (60%) were cured without any additional SUI procedure. Only two women (10%) required TVT for persistent grade 2 SUI. Two women (5.9%) developed de novo SUI, but none of them required an operation. RALLS repair for POP with mesh is safe and effective and may represent an alternative to sacral colpopexy/hysteropexy.

## Introduction

Pelvic organ prolapse (POP) is a frequent condition impairing women’s quality of life. The lifetime risk of undergoing POP surgery is estimated to be close to 20% [1]. Abdominal sacral colpopexy/hysteropexy (ASCP) is the gold standard for the treatment of apical vaginal and uterine prolapse [2]*.* During the last 2 decades, the developments of minimally invasive access by laparoscopy have reduced morbidity associated with transabdominal procedure [3]*.* More recently, access to robotic assistance made conversion from open to laparoscopic surgery more feasible without impairing results [4]. However, a difficult step for this procedure is the dissection of the sacral promontory to access the anterior longitudinal ligament where the mesh will be fixed. Although rare, sacrocolpopexy is associated with potentially serious complications such as life-threatening vascular injuries, ureteric damage, vertebral osteomyelitis, and nerve injuries resulting in chronic constipation and pain [5]. In 1967, Kapandji first described an alternative method to the ASC, which avoided the dissection of the sacral promontory and thus reduced its related risks. The procedure consisted in attaching the anterior vaginal wall and the uterine isthmus to the anterior–superior iliac spine with a mesh [6]. In the end of the nineties, the procedure was developed laparoscopically and modified with a higher and more lateral suspension [7, 8]. Dubuisson case series showed similar results to those of ASC, with an erosion rate of about 6% and a reoperation rate for recurrence close to 10% [8–10]. One of the weaknesses of these studies was the variability of surgical approaches including the use of various prostheses attached to the vaginal fascia with permanent sutures or with glue. Associated procedures were also not standardized sometimes using mesh to treat posterior defect, sometimes posterior colporrhaphy or McCall culdoplasty, with a large proportion of women having concomitant subtotal hysterectomy or preventive Burch colposuspension.

With the advances in robotic surgery, we performed the intervention using the da Vinci^®^ robotic system. From the start of our robotic experience, we standardized the procedure, thereby avoiding unnecessary hysterectomy and always used the same mesh and fixation technique. The robotic assistance allowed us to avoid the step of lateral transparietal passage of the dissector to pull up the lateral arms of the mesh reducing the number of scars [11]. The robotic assistance improved vision and ergonomics, reducing discomfort and fatigue of the surgeon.

The aim of this study was to analyze the anatomical and functional results of RALLS in a continuous series of women operated in our clinic. A secondary objective was to evaluate if a standardized technique using macroporous titanized polypropylene mesh and non-permanent sutures to fix it on the vaginal fascia may lower the erosion rate reported in previous laparoscopic studies.

## Materials and methods

**Fig. 1:**
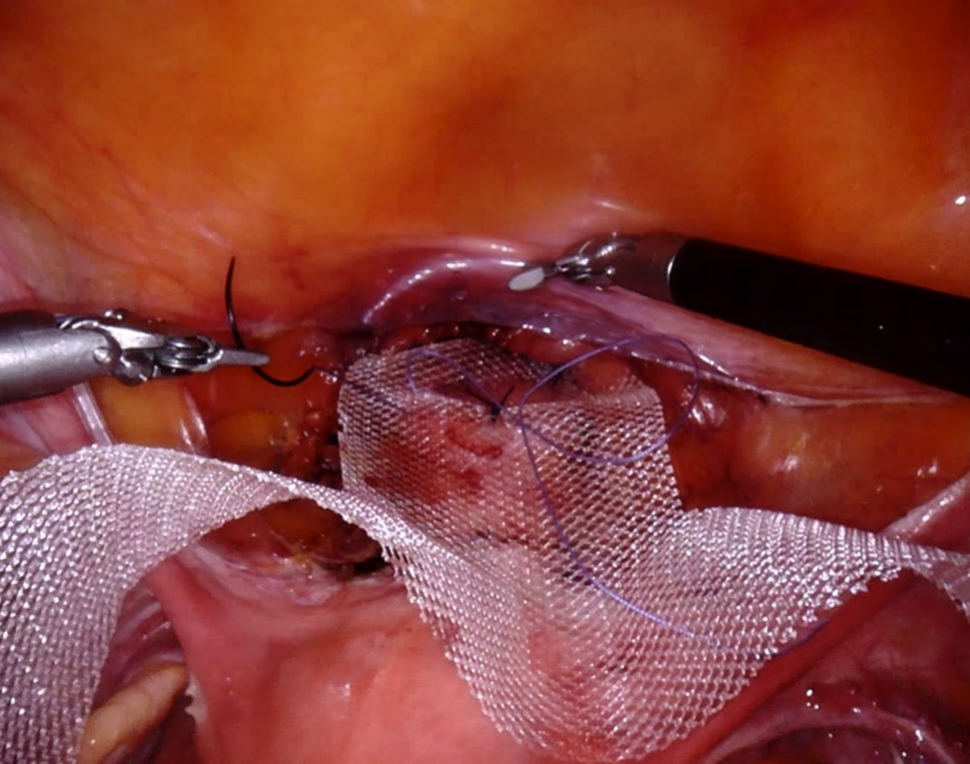
TiLOOP® titanised macroporous polypropylene mesh fixed to the vesicovaginal fascia with non-permanent sutures

**Fig. 2:**
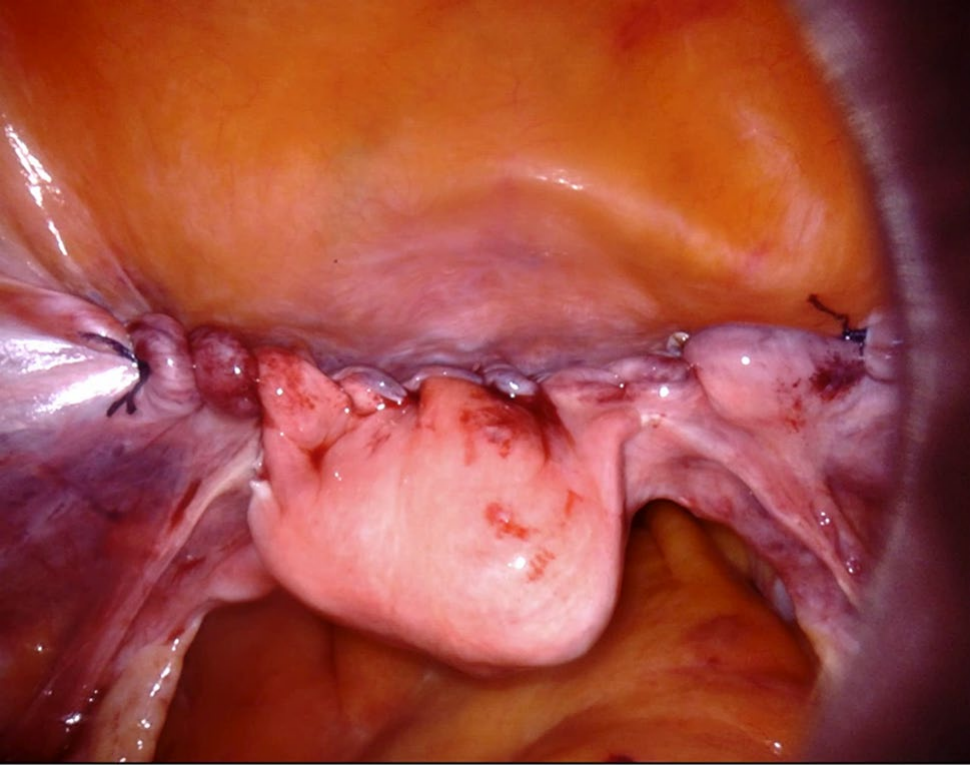
Final appearance after hysteropexy by robotically assisted laparoscopic lateral suspension

From March 2012 to January 2018, we performed RALLS repair with mesh in 59 consecutive women with symptomatic anterior vaginal wall and apical prolapse. The detailed technique is described and illustrated in our previous video article with a specific step-by-step procedure [11]. We consecutively used three da Vinci system (S, Si, and Xi by Intuitive surgical^®^) as they were progressively updated in our institution over the study period. The surgical procedure was the same with the three systems but the 12 mm umbilical trocar with da Vinci S and Si system became an 8 mm trocar with the Xi system. Docking was easier with the Xi system and we were able to displace the 10 mm paraumbilical assistant trocar to the suprapubic area. The two 8 mm lateral trocars were placed very laterally, 5 cm above the anterosuperior iliac spine. This allowed the assistant to pull up the lateral arms of the mesh through the trocars, avoiding supplementary incision and transparietal passage of the lateral arms of the mesh as it is performed in the standard laparoscopic technique. The very lateral position of the trocars was made feasible by robotic assistance. All patients were given preoperative prophylactic antibiotics (Mefoxitin—2 g intravenously) at induction of anesthesia. All patients were operated by a single surgeon (PD) experimented in laparoscopic surgery and trained in robotic surgery, assisted by residents in training. We used the same titanised macroporous polypropylene mesh for all patients (TiLOOP® “Prof Dubuisson”^®^ 9X 41.5 cm, 65 g/m^2^). The mesh was fixed to the vesicovaginal fascia (Fig. 1) with non-permanent sutures of 2–0 polyglactin 910 (Vicryl™ 2-0, JB needle, by Ethicon), and fixed to the anterior cervix and the isthmus uteri with two permanent polyester 0 sutures (Ethibon Excel™ 0, CT-1 needle by Ethicon). Peritoneum of the vesicovaginal fold was closed with a simple overlock of uninterrupted polyglactin 910 suture (Vicryl™ 0, CT-2 needle by Ethicon) (Fig. 2). All patients had postoperative fractional heparin. A vaginal swab with oestrogen cream (Oestogynaedron^®^) was placed in the vagina for the night and removed by next morning.

All women had preoperative urodynamics and POP assessment using the Pelvic Organ Prolapse Quantification system (POP-Q) [12]*.*They all had a postoperative 6 weeks assessment with clinical examination and a patient global impression of improvement (PGI-I) questionnaire for subjective evaluation of the procedure [13]. Between August 2017 and September 2019, all patients were contacted by two of the authors (SS and MA) by phone or letter to come to our clinic. We performed a clinical exam with POP-Q assessment and asked them to refill a patient global impression of improvement (PGI-I) questionnaire to evaluate long-term satisfaction with the procedure. Nowadays, many authors agree that clinically relevant POP are the ones overpassing the hymen, we used this definition for anatomical failure [14, 15]. Subjective failure was defined as PGI-I > 2.

This study was approved by the Institutional Ethics committee of the Geneva University Hospitals (protocol number 2017-00446). All patients gave their informed consent. We collected variables including age, weigh, height, parity, number of vaginal deliveries, menopausal status, the presence of diabetes, asthma, smoking, COPD, heart disease, constipation, or sexual activity, and history of a previous surgery for POP or urinary incontinence*.* Preoperative, perioperative, and longer term data were collected in case report forms for each patient. We systematically searched for complications such as mesh erosion, de novo SUI, reoperation for SUI or recurrent POP, and de novo dyspareunia.

We performed descriptive statistics. We used mean with standard deviation (SD), and the range to describe the general characteristics of our cohort*.* When more appropriate, we used median with interquartile (IQ) range. Medians were compared with Mann–Whitney test*.* *P* < 0.05 was considered to be statistically significant. Data analysis was performed using SPSS 25 statistical software (SPSS Inc., Chicago, IL, USA)*.*

## Results

POP repair was successfully completed in all 59 women without any perioperative complication. Five women were lost to follow-up. Two had moved abroad, and three had invalid addresses and phone numbers. Finally, 54 women of the cohort (91.5%) were available for analysis.

The characteristics of our study population are presented in Table 1. Most patients were postmenopausal and overweight. More than half had previous abdominal surgery, but none had previous hysterectomy. Seven patients (13.0%) had previous POP surgery (three anterior colporrhaphy and six posterior colporrhaphy) and one (1.9%) had previous operation for SUI (laparoscopic Burch colposuspension). Fifty-three patients (98.1%) had preoperative POP stage 3 or 4 and symptoms of UI were present in 50% of women. Preoperative urinary and fecal incontinence rate are presented in Table 2. The median operative time (from installation of uterine mobilization system to dressing of the wounds), was 180.0 min (IQ 155.0–204.8)*.* The median operative time of the robotic procedure was 114 min (IQ 99.3–129.0)*.* For the first twenty cases, the median operative time was 203.5 min (IQ 183.3–213.8), compared to 156.5 min (IQ 146.3–171.0) for the last twenty (*P* < 0.001). The median robotic time for the first twenty women was 126.5 min (IQ 106.8–135.8) compared to 110.0 (IQ 95.5–124.3) for the last twenty (*P* = 0.046). Perioperative characteristics are presented in Table 3. More than half of the patients had an associated surgical procedure, including six having a posterior colporrhaphy, but no concomitant SUI surgery. Mean estimated blood loss was 20.8 ml ± 55.6 (0–300 ml). There was no bladder, ureteral, or bowel injury, nor conversion in laparoscopy or laparotomy, and no supplementary trocar was added during the procedure. Mean VAS pain score was 2.8 (SD 1.9) and 1.9 (SD 1.7) on the first and second postoperative day, respectively. There was no postoperative fever or hematoma. Three women (5.6%) experienced urinary tract infection. The median length of postoperative stay was 2.5 days (IQ 2.0–3.0). Only one patient, known for a chronic cardiopathy, had a prolonged stay (15 days) for segmental pulmonary embolism.

**Table 1:** Characteristics of the study population (*N* = 54)

Characteristics	
Age (y) mean ± SD (range)	58.5 ± 10.4 (28.8–79.8)
Height (cm) mean ± SD (range)	163 ± 5.7 (151–174)
Weight (kg) mean ± SD (range))	71.2 ± 12.4 (49–107)
BMI (kg/m^2^) mean ± SD (range)	26.9 ± 4.6 (18.7–37.5)
BMI > 30 *n* (%)	13 (24.1)
Parity mean ± SD (range)	2.3 ± 1.0 (0–7)
Nulliparous *n* (%)	1 (1.9)
Primiparous *n* (%)	8 (14.8)
Multiparous (≥ 2 deliveries) *n* (%)	45 (83.3)
Menopause *n* (%)	43 (79.6)
Hormonal replacement therapy *n* (%)	13 (24.1)
Diabetes *n* (%)	11 (20.4)
Hypertension or cardiopathy	12 (22.2)
COPD	4 (7.4)
Smoking > 5 cig/day *n* (%)	10 (18.5)
Constipation *n* (%)	15 (27.8)
Sexual activity *n* (%)	33 (61.1)
Previous abdominal surgery	31 (57.4)
Previous POP surgery *n* (%)	7 (13.0)
Previous UI surgery *n* (%)	1 (1.9)

**Table 2:** Preoperative urinary or fecal incontinence of the study population

Characteristics	*N* = 54 (%)
Urinary incontinence	27 (50.0)
Pure stress urinary incontinence	10 (18.5)
Pure urge urinary incontinence	7 (13.0)
Mixed urinary incontinence	10 (18.5)
Stress urinary incontinence *n* (%)	20 (37.0)
Grade1	12 (22.2)
Grade 2	8 (14.8)
Grade 3	0
Occult stress incontinence at urodynamics *n* (%)	12 (22.2)
Fecal incontinence	4 (7.4)

**Table 3:** Perioperative characteristics (*N* = 54)

Characteristics	
Operative time (min) median IQ range	180.0 (155–204.8)
First twenty patients median IQ range	203.5 (183.3–213.8)
Last twenty patients median IQ range	156.5.0 (146.3–171.0)
Robotic time (min) median IQ range	114 (99.3–129.0)
First twenty patients median IQ range	126.5 (106.8–135.8)
Last twenty patients median IQ range	110.0 (95.5–124.3)
Docking time (min) median IQ range	8.0 (5.0–11.0)
First twenty patients median IQ range	8.0 (7.0–11.0)
Last twenty patients median IQ range	6.0 (4.3–10.0)
Concomitant procedures *n* (%)	
Adhesiolysis *n* (%)	16 (29.6)
Posterior colporrhaphy *n* (%)	6 (11.1)
Subtotal hysterectomy *n* (%)	1 (1.9)
SUI surgery *n* (%)	0
Bilateral salpingectomy *n* (%)	6 (11.1)
Estimated blood loss (ml) mean ± SD (range)	20.8 ± 55.6 (0–300)
Intraoperative complication *n* (%)	0
Conversion to laparoscopy or laparotomy *n* (%)	0

*IQ* interquartile, *SD* standard deviation

The mean follow-up was 33.6 ± 18.8 months (11.2–74.1). Anatomical results are presented in Tables 4 and 5. The objective cure rate (no prolapse beyond hymen in any compartment) was 98.1% at 6 weeks and 83.3% at long-term evaluation. The subjective cure rate (PGI-I ≤ 2) was 94.4% and 77.2% at short- and long-term follow-up, respectively (Table 6). Five women (9.3%) of the cohort required reoperation for POP recurrence. Two of them were operated before our study assessment. They had posterior compartment repair within the first year after the initial procedure. One woman was treated 10 months after first procedure for stage 2 rectocele by posterior colporrhaphy. The other patient had rectopexy and posterior mesh sacrocolpopexy 9 months after first operation for stage 3 rectocele and enterocele, with anal gas incontinence. Three women were operated during the months following our evaluation. One patient was treated 65 months after the first procedure for stage 3 rectocele and enterocele by vaginal repair. Two women presented with middle compartment recurrence with enlarged and long cervix, one of which had myomatous symptomatic uterus. Both women were treated by robotically assisted laparoscopic total hysterectomy with high uterosacral suspension of the vaginal vault, 31 and 54 months after the first procedure. There was no reoperation on the anterior compartment. However, we observed three cystoceles slightly overpassing the hymen margin, but all women were asymptomatic and did not require further surgical repair. Two women, one with stage 3 rectocele and one with stage 3 uterine prolapse, chose pessary instead of a reoperation. There was no mesh erosion at short- or long-term follow-up. Among the 20 women complaining of SUI preoperatively, 12 (60%) were cured at long-term follow-up. Two of them (10%), with preoperative grade 2 SUI, had TVT 12 and 46 months after the first operation, respectively. Two of the thirty-four other women (5.9%) developed de novo SUI, but none of them required SUI surgery. There were two women (3.7%) describing de novo dyspareunia at short- and long-term assessment. Eleven women (20.4%) presented constipation at short and long-term follow-up compared to fifteen (27.8%) preoperatively.

**Table 4:** Comparison between pre and postoperative POP (*N* = 54)

Stage	Preoperative	Early postoperative Mean 1.6 months	Late postoperative Mean 33.6 months
Stage 0	0	26 (48.1)	11 (20.4)
Stage 1	0	17 (31.5)	8 (14.1)
Stage 2			
Above hymen Beyond hymen	1(1.9) 0	10 (18.5) 1 (1.9)	26 (48.1) 2 (3.7)
Stage 3	51 (94.4)	0	7 (13.0)
Stage 4	2 (3.7)	0	0
POP beyond hymen	53 (98.1)	1 (1.9)	9 (16.7)

**Table 5:** Detailed anatomical results (*N* = 54)

Stage	Preoperative	Early postoperative (mean 1.6 months)	Late postoperative at (mean 33.6 months)
Anterior compartment (cystocele)			
0	0	50 (92.6)	32 (59.3)
1	3 (5.6)	2 (3.7)	4 (7.4)
2 above hymen 2 beyond hymen	2 (3.7) 3 (5.6)	2 (3.7) 0	15 (27.8) 2 (3.7)
3	45 (83.3)	0	1 (1.9)
4	1 (1.9)	0	0
Middle compartment (uterine prolapse)			
0	0	49 (90.7)	40 (74.1)
1	2 (3.7)	2 (3.7)	8 (14.8)
2 above hymen 2 beyond hymen	21 (38.9) 6 (11.1)	3 (5.6) 0	2 (3.7) 0
3	24 (44.4)	0	4 (7.4)^a^
4	1 (1.9)	0	0
Posterior compartment (rectocele)			
0	22 (38.6)	31 (57.4)	23 (42.6)
1	26 (48.1)	16 (29.6)	9 (16.7)
2 above hymen 2 beyond hymen	4 (7.4) 1 (1.9)	6 (11.1) 1 (1.9)	20 (37.0) 0
3	1 (1.9)	0	2 (3.7)^b^
4	0	0	0

Results are presented as *n* (%)

^a^One patient treated by pessary, two required laparoscopic total hysterectomy with high uterosacral suspension

^b^One enterocele treated with pessary, and one with vaginal repair

**Table 6:** Subjective outcome (Patient Global Impression of Improvement PGI-I)

PGI-I score	Early postoperative mean 1.6 months (*N* = 54)	Long term mean 33.6 months (*N* = 54)
PGI-I ≥ 2	51 (94.4)	44 (77.2)
1—Very much better	43 (79.6)	34 (63.0)
2—Much better	8 (14.8)	10 (18.5)
3—A little better	3 (5.6)	5 (9.3)
4—No change	0	3 (5.6)
5—A little worse	0	1 (1.9)
6—Much worse	0	1 (1.9)
7—Very much worse	0	0

Results are presented as *n* (%)

## Discussion

To our knowledge, this is the first reported series of RALLS with long-term outcomes. Our study confirms the feasibility and safety of this technique, and its effectiveness to treat anterior and apical POP with good objective and subjective long-term results. It allows preservation of the uterus which is important to many women [16]. Preserving uterus reduces operative time, mesh exposure, and blood loss without differences in POP recurrence [17]. Perioperative complications, including the risk associated with morcellation and the long-term risk of mesh erosion, are reduced by avoiding total hysterectomy [18–20]. LLS is well suited for hysteropexy and follows natural lateral attachment of the uterus. It maintains the vagina in a normal anatomical situation, which is not the case with sacrospinous hysteropexy, and to a lesser extent with sacrohysteropexy in which there is a slight right lateral traction on the mesh.

One of the objectives of this study was to evaluate if a standardized procedure using the same macroporous polypropylene mesh fixed to the vesicovaginal fascia only with non-permanent sutures would reduce the 6% risk of erosion described in previous LLS studies [8, 10]*.* With a mean follow-up close to three years, we observed no erosion. As the use of mesh is currently controversial, this is very reassuring information. Robotic assistance allows one to dissect the vesicovaginal space very precisely with almost no bleeding. It might participate to the absence of erosion. However, the type of mesh probably plays the most important role [21]. Mereu et al. in a recent 2 years follow–up, LLS series of 120 women using the same titanized polypropylene mesh observed only one erosion (0.8%). They do not describe the method of mesh fixation on the vagina but as they state that they followed the technique described by Dubuisson, they probably used permanent (polyester sutures). We believe that the use of permanent sutures such as polyester, used in previous laparoscopic LLS and LSCP studies, could increase the risk of vaginal erosion by conducting bacteria from the vagina to the mesh if the suture is too close to the vaginal mucosa. This was the case in Baines et al. recent study where vaginal exposure to polyester led to prosthetic erosions after LSCP [22]*.* Moreover, we believe the inflammatory reaction mediated by the mesh and neofibrosis to be responsible for the long-term support, thus rending permanent sutures unnecessary. We showed in a previous report that mesh type and posterior mesh placement represent risk factors for erosion [21]. Therefore, we decided not to place any mesh material in the posterior compartment. This strategy is sustained by the fact that the posterior compartment is best treated by vaginal access and does not require mesh material [23]*.* In this study, we decided to treat posterior defect only if it was symptomatic and discussed a two-step approach with patients to treat rectocele secondarily if necessary. Similar to Mereu et al. recent study, we often observed moderate asymptomatic rectocele during follow-up: 53.7% rectocele stage 1 or 2 not overpassing the hymen in our study compare to 18.3% in their study [24]*.* The difference may be due to their choice to exclude women with posterior defect to whom sacrocolpopexy was offered. However, in our study, only two patients required posterior compartment repair within the first postoperative year and only one over the following years of follow-up. We believe POP to be a functional pathology and it is important to treat only symptomatic prolapse not to do more harm than benefit to our patients. Moreover, posterior colporrhaphy is a simple procedure that can be done on an outpatient basis later if necessary. This strategy avoids the unnecessary use of mesh in the posterior compartment which is the case in standard sacrocolpopexy.

Mereu et al. reoperation rate for POP recurrence was 6.8% compared to 9.3% in our study. The difference is probably due to the selection of cases as previously discussed. If we exclude posterior compartment recurrence, the reoperation rate in the apical and anterior compartment drops down to 3.7% in our cohort. In comparison, in the Dubuisson 2011 series, the rate of reoperation for recurrence was 4.6% which was very similar. This rate is also similar to reoperation rates after LSCP and RALSCP. Sarlos et al. in a series of 101 LSCP patients treated with anterior and posterior mesh had a reoperation rate for recurrence of 3.5% at long-term follow-up. However, they experienced rather serious perioperative complications such as three rectal injuries, one with septic peritonitis, one with conversion in laparotomy, and one mechanical ileus also requiring laparotomy which is not the case with LLS or RALLS. In a systematic review of RALSCP by Serati et al., they described 3% (0–19) of intraoperative and 2% (0–8%) of severe postoperative complications, along with mesh erosion rates of 2% (0–8%) and a reoperation rate for recurrence of 3.3% [25]. In a large LSCP review with more than 1000 patients with a mean follow-up of 2 years, they had a 6.2% reoperation rate for POP recurrence [26]*.* In a more recent French LSC series of 464 women with a mean follow-up of 4 years, the reoperation rate for POP recurrence was 5.1% and mesh related reoperation rate was 2.8% [27]*.* Among our reoperations for POP recurrence, two women presented with hypertrophic and elongated cervix and required hysterectomy with high uterosacral suspension. Both already had hypertrophic cervix before first intervention and we believe it might represent a risk factor for failure with this technique*.*

In case of SUI or occult SUI at urodynamics, we used a two-step strategy. Our patients agreed on treating them secondarily only if SUI was still bothering them after POP repair. This strategy is supported by current medical literature [28, 29]*.* An interesting and unexpected result of our study was the large proportion (60%) of cure of preoperative SUI. A similar improvement of preoperative SUI without concomitant SUI surgery was also observed in a recent LSCP study [30]. We hypothesize that an anterior vaginal mesh, by lifting up vesicovaginal fascia, may improve suburethral hammock suspension. We observed only two cases (5.9%) of de novo SUI which is close to the rate of 2.5% found in a recent LLS study [24].

In the only other published RLLS series, Simoncini et al. reproduced each step of the standard LLS making a 3 mm skin incision 2 cm above and 2 cm laterally to the anterior superior iliac spine [31]*.* As described in our first report of the technique, we believe it is unnecessary with robotic assistance [11]*.* Indeed, an interesting benefit of robotic surgery is the fact that with the help of computerized assistance, it is possible for the surgeon to introduce trocars on the abdominal wall that are ergonomically inaccessible in standard laparoscopy. We placed our lateral trocars very laterally to be able to pull the arms of the mesh through the same abdominal wall incisions. By avoiding the step of the transparietal passage of the laparoscopic forceps used to pull the lateral arms of the mesh, it allowed us to reduce the number of incisions of the standard LLS technique. We believe that apart from aesthetic considerations, it may also potentially reduce postoperative pain. It may also help avoid potential damage to abdominal wall nerve of the lumbar plexus, in particular the iliohypogastric and ilioinguinal nerves that run between the muscles of the abdominal wall. To fix the mesh, Simoncini et al. used three rows of sutures, using long-term absorbable 2–0 monofilament suture for the first two rows (Maxon^®^), and a third row of non-absorbable 2–0 sutures (Prolene® Ethicon) on the upper part around the cervix. One patient presented immediate extrusion of one suture and required removal of underlying mesh. As discussed earlier, we believe that short-term absorbable suture is better suited than long-term or permanent sutures to fix the mesh to the vesicovaginal fascia. It reduces the risk of erosion without impairing results. Fibrosis will form and fix the mesh in the very first postoperative days.

Another data highlighted by our study is the learning curve necessary to improve efficiency of RALLS. In comparing the first twenty and last twenty procedures, we observed a significant reduction in the median total operative and console time. From our experience we estimate that 25–30 procedures are required to reduce and stabilize console time and total surgery time which is quite similar to the learning curve of RALSCP [32].

The limitation of our study is that it is the experience of a single institution with a single surgeon, without any control group, which limits generalization of the results. The number of patients is small and larger series are required to confirm and disseminate the technique to other centres.

The strength of our study is to provide a standardized series initiated from the beginning of our robotic experience with a long-term follow-up and the availability of precise preoperative, perioperative, and postoperative data. Our robotic program included a continuously updated computerized register which allowed us to collect specific perioperative data.

## Conclusions

RALLS POP repair with mesh is a safe and effective technique for the treatment of anterior vaginal wall and uterine prolapse, and is often associated with improvement of SUI. Anatomical and functional results are similar to the standard LLS technique. By avoiding promontory dissection, RALLS may have several advantages over RALSCP by reducing perioperative risks such as bowel, vascular and nerve injuries as well as osteomyelitis. It may represent a useful alternative in cases of pelvic kidney, bony abnormalities, and difficult promontory dissection such as fatty presacral space with difficulties reaching the longitudinal ligament. In case of a concomitant posterior defect, secondary worsening is possible and a standard colporrhaphy made at the same time or in a second step should be discussed with the patient.

## Abbreviations

ASCP: Abdominal sacral colpopexy
LSCP: Laparoscopic sacrocolpopexy
RALSCP: Robotically assisted laparoscopic sacrocolpopexy
POP: Pelvic organ prolapse
POP-Q: Pelvic organ prolapse quantification system
LLS: Laparoscopic lateral suspension
RALLS: Robotically assisted laparoscopic lateral suspension
TVT: Tension-free vaginal tape
BMI: Body mass index
HT: Hormonal replacement therapy
UI: Urinary incontinence
SUI: Stress urinary incontinence
UUI: Urge urinary incontinence
MUI: Mixed urinary incontinence
IQ: Interquartile
TH: Total hysterectomy
SH: Subtotal hysterectomy
VAS: Visual analogue scale
COPD: Chronic obstructive pulmonary disease
